# Role of the Amygdala in the Complex Relationship Between Sleep and Anxiety

**DOI:** 10.3390/ijms27177814

**Published:** 2026-08-31

**Authors:** Filip Grzybowski, Piotr Kaczmarski, Dominik Strzelecki, Adam Wysokiński, Piotr Białasiewicz, Agata Gabryelska

**Affiliations:** 1 Department of Sleeping Medicine and Metabolic Disorders, Medical University of Lodz, 92-215 Lodz, Poland; 2 Department of Affective and Psychotic Disorders, Medical University of Lodz, 92-216 Lodz, Poland; 3 Department of Old Age Psychiatry and Psychotic Disorders, Medical University of Lodz, 92-216 Lodz, Poland

**Keywords:** sleep, anxiety, amygdala, sleep disorders, GABA, REM, NREM

## Abstract

This review explores the multifaceted roles of the amygdala, focusing on its structural components, projections, physiological aspects, and key functions in emotional processing and fear conditioning. Beyond traditional roles, it examines the amygdala’s involvement in sleep architecture during the NREM and REM phases. In NREM sleep, the amygdala is involved in memory replay and encoding. During REM sleep, it is highly active, contributing to the emotional content of dreams. This state is characterized by increased cerebral blood flow to the amygdaloid complexes and the generation of Ponto–Geniculo–Occipital (PGO) waves, which are linked to vivid visual imagery and emotional experiences. The review establishes a critical link between the amygdala and the cycle of anxiety disorders and sleep disturbances. It explores anxiety disorders’ specific impact on the amygdala and their influence on sleep patterns. Additionally, the review discusses how medications for anxiety disorders and sleep disturbances act on the amygdala. Selective serotonin reuptake inhibitors (SSRIs) regulate amygdala function, affecting emotional responses and connectivity patterns, improving anxiety, and potentially enhancing sleep quality over time. Trazodone, a serotonin antagonist and reuptake inhibitor (SARI), improves sleep by blocking serotonin receptors and is effective in treating post-traumatic stress disorder (PTSD) related sleep disturbances. Benzodiazepines and zolpidem affect sleep architecture and anxiety by selectively enhancing the actions of both endogenous and exogenous GABA through GABA-A receptors. At the same time, agomelatine, an antidepressant with anxiolytic properties, improves sleep quality without disrupting REM sleep and treats insomnia associated with various conditions, including alcohol dependence. The aim of this review is to summarize amygdala functions, with a focus on their role in regulating sleep and affective responses. Moreover, it discusses possible therapeutic targets in the amygdala for anxiety and sleep disorders.

## 1. Introduction

The amygdala, situated intricately within the temporal lobe, has long been recognized for its multifaceted functions, ranging from emotion regulation to social interactions. While renowned for its role in anxiety generation and associated psychiatric conditions, emerging research underscores its pivotal role in orchestrating sleep. Given the profound comorbidity between anxiety disorders and sleep disturbances, we posit that unraveling the nuances of amygdala function is imperative for a comprehensive understanding of these phenomena.

This review examines the anatomy and physiology of the amygdala, elucidates its role in sleep regulation, and explores its connection to anxiety disorders. We summarize the current literature, including experimental studies and reviews, on the potential clinical implications of disrupted amygdala function and highlight promising therapeutic targets in the amygdala.

We conducted a literature search using PubMed, Google Scholar, and Scopus. The terms used included: “amygdala physiology”, “amygdala neurotransmitters”, “amygdala functions”, “amygdala and sleep”, “amygdala and sleep architecture”, “amygdala and sleep disorders”, “amygdala and anxiety disorders”, “sleep disorders and anxiety disorders”, “amygdala and anxiolytic drugs”, “amygdala and hypnotics”. Additionally, we used citation chaining to broaden the scope of the review. The search was conducted in 2025 and included original research articles, review articles, case studies, reports, and book chapters. We did not limit the literature search to a specific time range.

## 2. Amygdala Overview

The term amygdala, originating from Greek and meaning “almond”, was first introduced by the German physiologist Karl Friedrich Burdach in 1822 [1]. It is a limbic structure located deep in the temporal lobe of the human brain, recognizable as a bump in the base of the caudal cerebral hemispheres, a feature targeted by the lateral olfactory tract [2].

### 2.1. Structure of the Amygdala

The amygdala is composed of about 13 nuclei, which can be divided into three main groups. The basolateral amygdala (BLA) includes the basal nucleus, the lateral nucleus, and the accessory basal nucleus. The superficial (SF) amygdala comprises the cortical nuclei and the nucleus of the lateral olfactory tract. The centromedial group is composed of the medial (MA) and central amygdala nuclei (CeA). Moreover, there is a distinct set of nuclei that cannot be easily classified into the three groups and are listed separately; these include the intercalated cell masses and the amygdalohippocampal area. This division, although sometimes questioned, seems important, as these groups differ in their roles, histochemistry, and structure [3]. The basolateral amygdala (BLA) is primarily involved in fear conditioning [4], multimodal sensory integration [5], and avoidance behavior [6]. The superficial group contributes to social signal detection and emotional responses [7], while the centromedial nuclei regulate prediction error signaling, learning, reward processing, and autonomic responses [8,9]. The intercalated cell masses mediate inhibitory control between nuclei, supporting emotional learning [10], and the amygdalohippocampal area is associated with sociosexual behaviors and metabolic or cognitive functions [11]. A summary of the main functions of these amygdala groups is presented in Table 1.

The connections between the amygdala and other brain regions are numerous, encompassing a vast array of efferent and afferent neurons that participate in this intricate network. Inputs to the amygdala can be categorized into two groups: those originating from cortical and thalamic structures, and those originating from the hypothalamus or brainstem. Cortical and thalamic inputs convey information from sensory areas and structures associated with memory systems, whereas hypothalamic and brainstem inputs originate from regions involved in behavior and autonomic systems [3]. The developed system of efferent amygdala projections is crucial for eliciting appropriate emotional responses to diverse stimuli. Efferent fibers from the amygdala originate mainly in the BLA and CeA and connect these structures with the cortex, hypothalamus, and other limbic structures [12]. Above that, the amygdala has a rich system of intra-amygdaloid connections, enabling communication between different parts of this organ [3].

**Figure 1:**
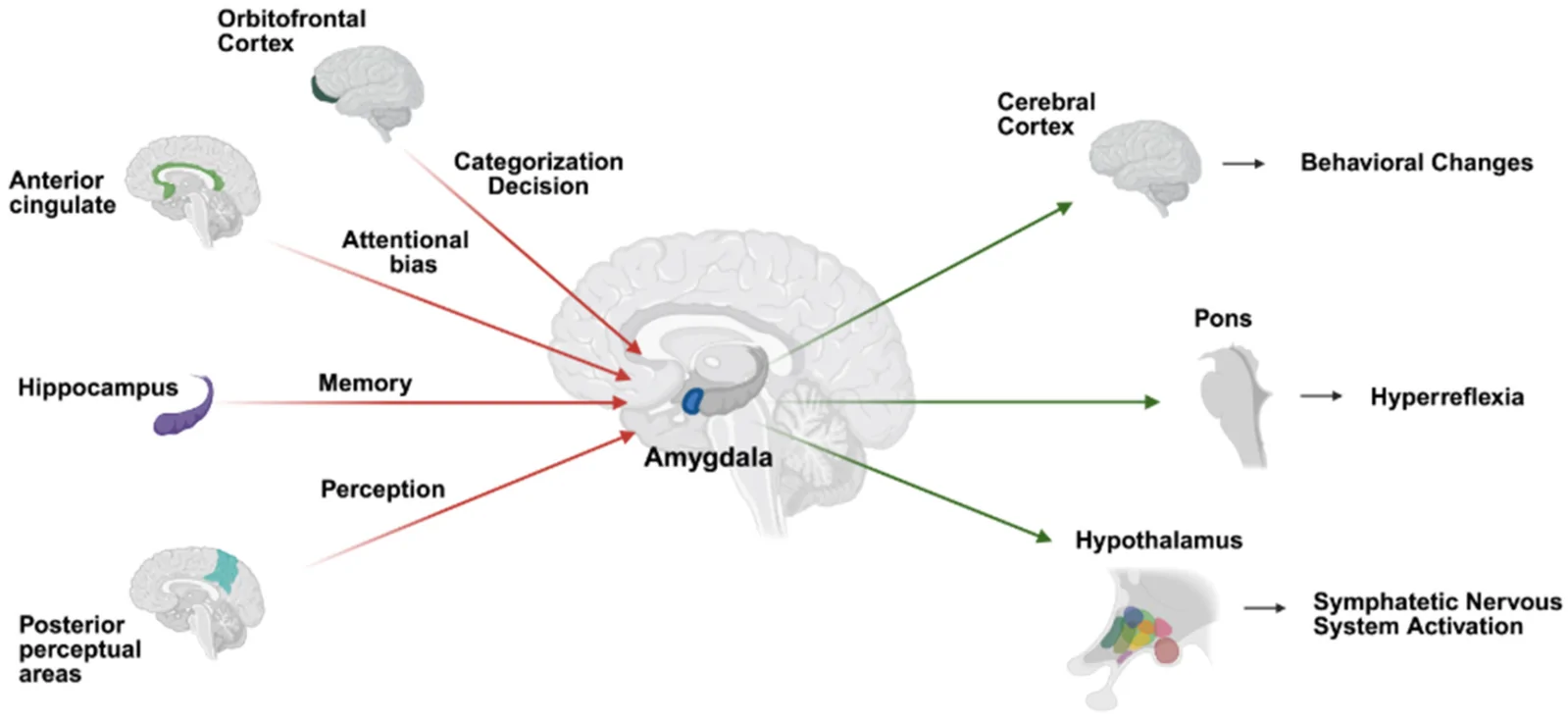
Physiology of the Amygdala. The amygdala operates within a broad network of afferent (red arrows) and efferent (green arrows) connections, receiving input primarily from the basolateral amygdala (BLA), its primary input station. Signals from the hippocampus, posterior perceptual areas, anterior cingulate cortex (ACC), and orbitofrontal cortex reach the amygdala, each contributing specific types of information. Posterior perceptual areas provide direct representations of objects or events, while the ACC modulates attentional bias. The hippocampus influences memory processes relevant to anxiety, and the orbitofrontal cortex contributes to decision-making, categorization, and emotion regulation. After intra-amygdaloid processing, the amygdala determines the anxiety response, activating efferent pathways that lead to autonomic dominance, hyperreflexia, and behavioral changes characteristic of anxiety.

Available data suggest that the fundamental model of information flow in the amygdala operates as follows: information enters the amygdala through the BLA group of nuclei, undergoes local processing, and subsequently proceeds to the CeA nuclei, which serve as the output station [13]. Figure 1 presents a simplified version of amygdala function.

### 2.2. Gamma-Aminobutyric Acid and Its Receptors in the Amygdala

The cells of the amygdala present a variety of diverse receptors for different neurotransmitters. GABA (gamma-aminobutyric acid) receptors are predominantly expressed in amygdala cells. These receptors play a significant role in inhibitory neurotransmission, regulating the excitability and overall function of the neural circuits in this brain region [14]. Research suggests that individuals with anxiety disorders may have abnormalities in GABAergic neurotransmission [15]. The proper functioning of GABA receptors in the amygdala helps in reducing anxiety-related behaviors. Low GABA levels or impaired GABA receptor functioning can lead to increased neuronal excitability, contributing to anxiety symptoms [16]. Another process in which GABAergic neurons in the amygdala play a critical role is fear extinction, defined as the inhibition of fear responses to stimuli that were previously associated with fear. Impairment in this mechanism is also thought to be involved in the pathogenesis of anxiety disorders [17].

### 2.3. Glutamate and Its Receptors in the Amygdala

The opposing neuromodulator, glutamate, acts on this structure through AMPA (Alpha-Amino-3-hydroxy-5-methyl-4-isoxazolepropionic Acid), NMDA (N-Methyl-D-aspartate), and kainate receptors, as well as eight subtypes of metabotropic receptors. Enhanced glutamatergic transmission is widely recognized as a pivotal mechanism underlying amygdala recruitment during acute stress [18,19]. Even though glutamate is used to activate the amygdala, agonists of specific glutamate metabotropic receptors (mGlu2, mGlu3) have shown efficacy in the treatment of generalized anxiety disorder [20]. Moreover, both systemic and intra-amygdalar infusions of D-Cycloserine, a partial NMDA receptor agonist, led to dose-dependent facilitation of fear extinction in rats [21]. These data demonstrate the amygdala’s crucial role in emotion generation.

### 2.4. Serotonin and Its Receptors in the Amygdala

Another important substance involved in processes taking place in the amygdala is serotonin. Serotonin exerts its influence on the amygdala through various serotonin receptors, including 5-HT1A, 5-HT2A, and 5-HT3 receptors. Activation of these receptors can either inhibit or excite amygdala neurons, depending on the specific receptor subtype and the neuronal circuit involved. For instance, activation of 5-HT1A receptors generally inhibits amygdala activity, leading to anxiolytic effects, while stimulation of 5-HT2A receptors can enhance excitatory neurotransmission, potentially contributing to anxiety and fear responses [22]. Serotonin primarily influences the amygdala through interneurons, whose activation modulates the release of other neurotransmitters, such as GABA. Additionally, research data suggest that serotonin plays a crucial role in processes such as synaptic plasticity and fear memory processing [23].

### 2.5. Dopamine Receptors in the Amygdala

Other receptors are also involved in regulating the amygdala. For instance, the dopamine D1 receptor (D1) is probably responsible for enhancing the amygdala response when sensory input is present [15,24]. In a study conducted by Takahashi et al., functional magnetic resonance imaging (fMRI) was employed to investigate healthy volunteers. The study employed multimodal voxel-wise correlation analysis to examine the relationship between the fMRI signal and D1 binding, as measured via positron emission tomography (PET). Results revealed that D1 binding in the amygdala positively correlated with changes in amygdala signal in response to fearful faces. In reaction to stress, dopamine release in the amygdala escalates [24]. Moreover, animal studies have demonstrated that dopamine enhances the amygdala response by intensifying excitatory sensory input and decreasing inhibitory input from the prefrontal cortex [25,26]. Dopamine release in the amygdala escalates the stress response; thus, it can be considered an important survival mechanism by activating the autonomic nervous system, which is needed when approaching dangerous situations.

### 2.6. Opioids and Their Receptors in the Amygdala

Another group of receptors involved in amygdala function is the opioid receptor family. It has been reported that the kappa opioid receptor in the CeA modulates nociceptive processing by acting on amygdala corticotropin-releasing neurons [27]. Given its significance in associative learning and the affective dimension of pain, the amygdala is also targeted by opioid drugs [28]. They act at several sites within this structure, including by inhibiting half of the CeA neurons [29]. Orexin is also involved in fear modulation, as fear-conditioned orexin receptor knockout mice show decreased fear expression, and orexin receptor antagonists inhibit fear acquisition and expression. Elevated orexin signaling in the amygdalo–cortical–hippocampal circuit enhances avoidance behaviors. Orexin also plays a key role in stimulating sympathetic nervous system activity and triggering stress responses through the hypothalamus–pituitary–adrenal (HPA) axis. Notably, recent studies have shown that increased orexin activity promotes wakefulness, and an orexin antagonist enhances sleep onset and maintenance [30].

### 2.7. Functions of the Amygdala

The primary function of the amygdala is fear processing. Various neuroimaging techniques, such as fMRI or PET, have shown that regional blood flow in the amygdala is significantly increased during fearful situations [31,32,33]. Moreover, ablation of the CeA and BLA impairs fear learning in rodents [34]. The widely accepted model of amygdala function begins with the input system, which incorporates pathways from the cortex, hippocampus, limbic system, and sensory thalamus. The subsequent step involves the intramygdaloid neurons, where decisions about the appropriate reaction are made. When the perceived level of danger is sufficiently high to provoke fear, the sympathetic system is activated, leading to changes such as elevated heart rate and blood pressure, bradycardia, or the release of corticosteroids. Moreover, behaviors like active avoidance or freezing are also promoted [35].

Nevertheless, the role of the amygdala does not terminate here. A significant number of studies suggest that the amygdala also plays a vital role in processes such as decision-making, motivational behaviors, and learning [36,37,38]. This structure is also important in social interaction. In a study conducted by Kuga et al., the researchers observed an increase in power of 4–7 Hz oscillations and a decrease in power at 30–60 Hz in the BLA of rodents when they were required to attend to another target mouse [39]. Furthermore, another report showed that the posteromedial nucleus of the cortical amygdala was connected with the suppression of male mating when a female is sick [40].

Important insights into amygdala function can be drawn from the case study of patient SM, whose amygdala was bilaterally damaged due to the rare Urbach–Wiethe Disease. The case presentation showed the amygdala’s inability to condition to aversive stimuli [41], to recognize fearful faces [42], or to activate fear during exposure to various fear-provoking stimuli [43]. Additionally, the amygdala has been shown to directly detect changes in CO_2_ and pH via acid-sensing ion channels, resulting in CO_2_-evoked fear responses that occur even in patients with similar bilateral amygdala damage [44]. These findings, combined with observations from neurosurgical experiments involving direct amygdala activation [45], have led to the development of the Apnea-Induced Anxiety model. This model suggests that repeated episodes of apnea could be unconsciously triggered by amygdala activation, resulting in a temporary increase in CO_2_ level that evokes fear and anxiety [46]. As anxious patients tend to be hypersensitive to CO_2_, even short or weak activation of the amygdala can lead to an excessive anxiety experience. Moreover, under this hypothesis, behavioral patterns such as avoidance may serve as a safety mechanism to prevent an increase in CO_2_.

## 3. Involvement of the Amygdala in Sleep

An increasing number of studies demonstrate that the amygdala is also involved in different aspects of sleep regulation. Sleep is commonly defined as a rapidly reversible state of immobility and significantly reduced sensory responsiveness. Human sleep is composed of two distinct brain activity states, NREM (Non-Rapid Eye Movement) and REM (Rapid Eye Movement), which occur in a cyclical pattern and play complementary roles in recovery after waking. An average first sleep cycle lasts 70–100 min, in contrast to the subsequent 90–120 min cycles. Sleep architecture is typically assessed using electroencephalography (EEG). This diagnostic procedure measures the brain’s electrical activity by placing electrodes on the subject’s scalp, as each sleep stage manifests as distinct fluctuations in brain activity [47].

### 3.1. NREM Sleep

NREM is the initial phase of each sleep cycle, and according to the American Academy of Sleep Medicine, it comprises three stages (N1, N2, N3) [48]. Overall, NREM sleep constitutes approximately 75% of nightly sleep; however, the NREM phase shortens with each cycle, whereas REM sleep elongates [49]. Stage N1 is typically characterized by the disappearance of the alpha rhythm and the onset of roving eye movements. EEG readings during this stage reveal medium-amplitude, mixed-frequency activity primarily in the 4–7 Hz range, accompanied by irregularly spaced bursts of slow waves. Notably, vertex sharp transients (VST) emerge, which are sharply contoured, synchronous waves with the highest amplitude over the central derivations [50]. These VSTs typically last for less than 0.5 s and may exhibit varying amplitudes on either side. Additionally, positive occipital sharp transients of sleep appear, characterized by either mono- or biphasic positive triangular waves that are most pronounced in the occipital regions of the head. Stage N2 is characterized by bilaterally synchronous theta activity (>3.5 to <8 Hz frequency, amplitude greater than 30 µV), often accompanied by the presence of sleep spindles or K-complexes, or both. K-complexes are identified by a distinctive pattern of a sharp negative wave immediately followed by a positive wave (V-shaped), standing out from the background EEG. These complexes typically last for 0.5 s and are most prominent in the fronto-central derivations. Sleep spindles, on the other hand, are defined as distinct 12–14 Hz waves, with a typical range of 11–16 Hz (most commonly 12–14 Hz). They typically last 0.5 s or longer and reach maximal amplitude in the central derivations. Stage N3 is characterized by high-amplitude delta waves in the range of 0.5 to 2 Hz, with amplitudes reaching approximately 75 μV, as measured over fronto-central derivations. Although K-complexes and sleep spindles may still be present, occurrences of positive occipital sharp transients of sleep (POSTs) are rare during this stage. N3 sleep is typically scored if delta slowing is observed in at least 20% of the epoch. It primarily occurs during the initial one-third of the night, and this is clinically significant as NREM parasomnias, such as sleepwalking and night terrors, are commonly observed during this period [51]. Physiological changes during the NREM sleep phase include decreased sympathetic nerve activity, hypoventilation, and altered endocrine gland secretion [52].

### 3.2. Role of the Amygdala in NREM Sleep

While the amygdala is extensively studied during REM sleep, researchers are also actively exploring its role in NREM sleep. A study by Muñoz-Torres et al. showed that hippocampal–cortical synchrony at sleep spindle frequencies is highest during this phase [53]. As synchrony may reflect the specific interrelation of brain regions, we can hypothesize that the amygdaloid complex may influence specific memory-related processes. This hypothesis is grounded in findings that emotionally significant experiences are remembered better than neutral ones [54], and that memory stabilization occurs primarily during NREM sleep [55]. Additionally, studies have identified delta (0.5–4 Hz) and sigma (12–16 Hz) activity in the amygdala during the N3 phase of sleep [51].

NREM sleep also involves sharp wave ripples, marked by large-amplitude, 40–100 ms-long detections, that are often followed by rapid oscillations. While predominantly observed in specific areas of the hippocampus, similar events occur in the amygdala [56].

Sharp wave ripples are thought to contribute to memory replay during NREM sleep, as the firing patterns of neurons surrounding these events closely mirror temporally compressed versions of patterns observed during recent learning in rats [57]. These findings suggest its potential involvement in extensive functional networks, similar to those associated with the hippocampus, thereby facilitating information transfer between structures [58]. Furthermore, increased functional connectivity among the cerebellum, hippocampus, parahippocampus, and amygdala, observed in both animal and human neuronal recordings, also suggests crosstalk in N3, probably facilitating encoding processes [59,60]. In summary, the role of the amygdala during NREM sleep remains intriguing, as research suggests its involvement in memory processes. Ongoing studies aim to elucidate its complex influence during this phase.

### 3.3. REM Sleep

To maintain proper sleep architecture, NREM sleep must transition to the next phase, REM sleep, at the appropriate time. While the exact mechanisms underlying this phase transition are not fully understood, the structure in question plays a role in this process. A study by Hagesawa et al. found that a transient increase in dopamine in the BLA of the amygdala terminates NREM sleep and initiates REM sleep [61]. This process appears to be mediated by serotonin, as inhibiting 5-HT neurons in this region negates the effect [62].

During REM sleep, brain activity exhibits EEG patterns similar to those present during the awake state. This stage shows low voltage and mixed EEG frequency [51]. Brain waves are fast and desynchronized, sometimes described as sawtooth-like. This stage could be subdivided into phasic and tonic phases. During the phasic phase of REM sleep, distinct features such as eye movements, middle ear muscle contractions, and an irregular heart rate are observed, indicating intense dreaming [63]. In contrast, the tonic phase, although showing EEG activation, results in lowered arousal thresholds and different processing of external stimuli. Dreams can occur in both phases, but they tend to be less vivid and more abstract during the tonic phase, which is marked by the appearance of alpha- and beta-frequency bands in the EEG [64].

### 3.4. Role of the Amygdala in REM Sleep

The precise function of REM sleep remains elusive, yet numerous hypotheses have been proposed in scientific discourse. In the context of this review, the most important is one proposing its role in amygdala-related memory processing [65]. Some researchers suggest that memory consolidation processes associated with REM sleep likely involve the modulation of amygdala-related networks, potentially adjusting the strength of emotional responses, such as reinforcing or diminishing negative emotions and fear, in response to the organism’s adaptive needs [66]. This hypothesis is known as sleep-to-remember, sleep-to-forget [67]. The neurobiological processes underlying REM promote the dissociation of memory’s emotional content from its associated arousal. This separation enables disparate processes of attenuation and augmentation, involving both the erosion of the memory’s emotional valence and the concurrent reinforcement of its factual content [68]. The formation of episodic memories during wakefulness involves the coordinated encoding of information in the hippocampus, facilitated by the amygdala and modulated by elevated levels of aminergic neurotransmission [69]. Infusing beta-adrenergic receptor agonists into the BLA enhances memory consolidation, while a post-training infusion of beta-blockers blocks this effect [70]. Glucocorticoids also play a role in this process, as infusing glucocorticoid receptor agonists into the BLA and hippocampus enhances memory consolidation [71]. During subsequent REM sleep, the same neural structures seem to be reactivated. This phenomenon enables the reprocessing of previously learned emotional experiences. However, this occurs in a neurochemical environment devoid of aminergic modulation, dominated instead by cholinergic neurochemistry [72]. Consequently, emotional memory reprocessing reduces the affective tone initially associated with the encoded event, concomitant with a progressive neocortical consolidation of the information. At the same time, some researchers propose that this process might be implicated in the pathogenesis of post-traumatic stress disorder (PTSD). Attempts to establish a definitive association between these mechanisms and the disorder have been inconclusive so far [68,73]. Furthermore, REM is also likely involved in brain maturation as it provides the necessary stimulation for the initial development and survival of sensorimotor neuronal networks [74]. Li et al. used a motor learning model in 3-week-old mice and demonstrated that REM sleep promotes the formation and maintenance of synapses. They found that REM sleep facilitated the formation of new spines in the mouse motor cortex during both development and after a motor learning task. Additionally, REM sleep strengthened the synapses that had already formed, thereby enhancing performance following motor learning [75].

Such findings can be explained by the upregulation of some synaptogenic factors (e.g., brain-derived neurotrophic factor (BDNF)) during REM. On the electrophysiological level, REM is also beneficial, as it provides a specific environment needed for proper neurodevelopment [76]. Synchronized neural oscillations stimulate the sensorimotor cortices and distant structures, such as the brainstem and hippocampus [77].

#### 3.4.1. Amygdala and Emotional Learning

Emotional learning refers to the process through which individuals acquire, understand, and manage emotions. It involves the ability to recognize, interpret, and respond to emotional cues in oneself and others. An integral component of this process, fear memory consolidation, was highly associated with REM-specific events. In fact, after fear conditioning, the responsiveness of amygdala and thalamic neurons to conditioned stimuli is enhanced during REM sleep. The study by Hennevin, Maho, and Hars investigated whether neurons in the lateral amygdala (LA) and medial geniculate (MG), pivotal in auditory fear conditioning, exhibit physiological plasticity during REM sleep. Results show that conditioned responses in both LA and MG rapidly emerge, have short latency, and persist during PS, suggesting the retained expression of learning-induced plasticity during this phase of sleep [78]. Furthermore, aversive events have been shown to increase REM sleep duration in rats [79], whereas REM deprivation after training impairs the consolidation of aversive memories [80]. Intriguingly, recent studies have delved deeper into changes in amygdala electrical activity, shedding light on the neural processes underlying fear conditioning during REM sleep. Theta activity refers to a specific rhythmic neural oscillation occurring at a frequency range of about 4–8 Hz. This pattern is present in the amygdala–hippocampal circuit and is believed to play a role in consolidating the aforementioned fear memory [81]. Theta coherence, REM sleep EEG theta amplitude, and phasic pontine waves (P-waves, pontine component of PGO waves) may be more accurate predictors of successful consolidation of fear memory than the amount of REM sleep [82]. Moreover, a study conducted by Machida et al. found that optogenetic inhibition of glutamatergic cells in the BLA reduced EEG theta activity, whereas inhibition of these neurons increased it [83]. As these processes were not associated with changes in other sleep parameters, we can hypothesize that these cells are specifically responsible for controlling the generation of the theta rhythm.

#### 3.4.2. Amygdala and Dreaming

**Figure 2:**
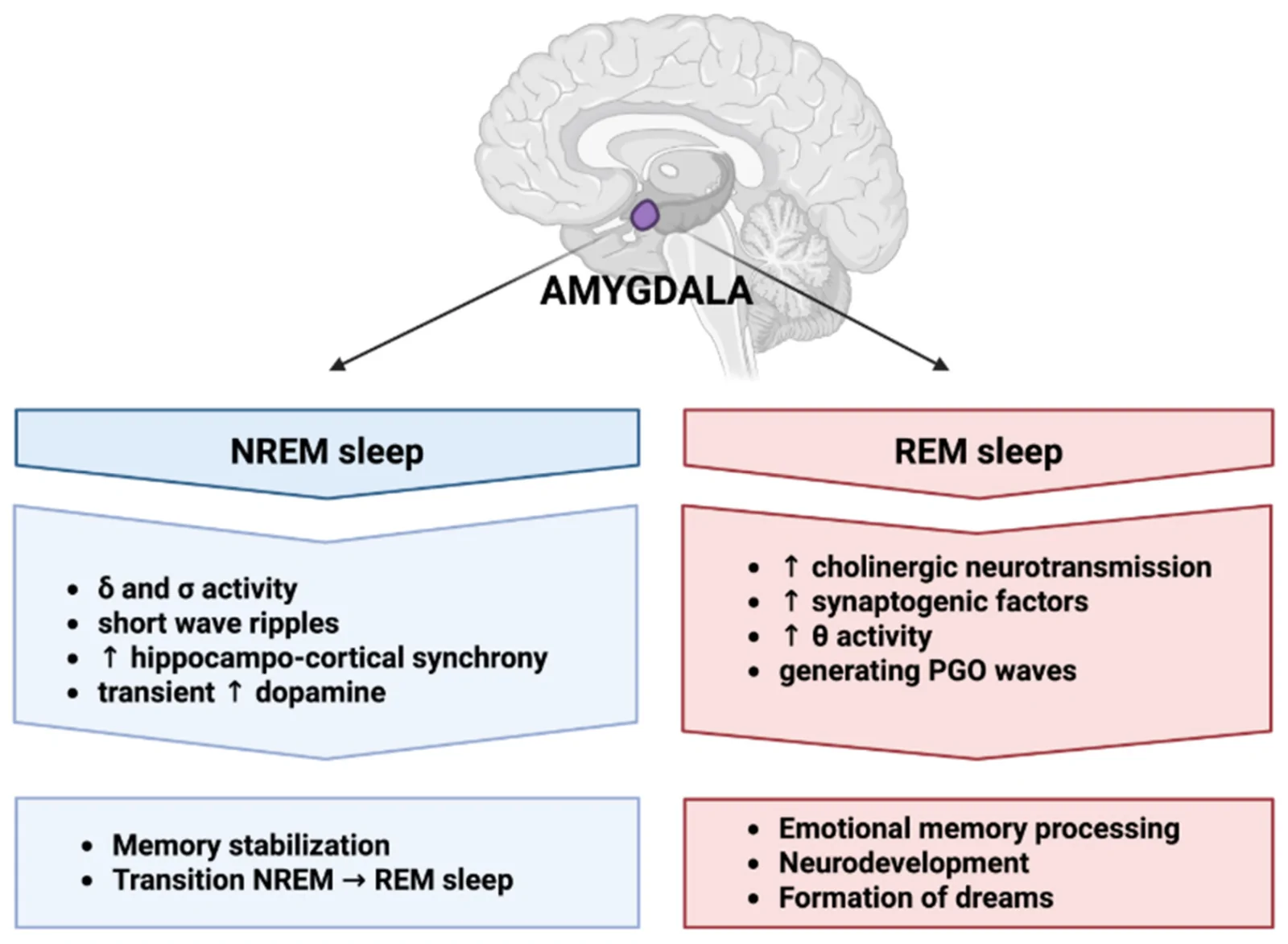
Role of the amygdala in rapid eye movement (REM) and non-rapid eye movement (NREM) sleep. Changes in amygdala function during specific sleep phases determine its role in physiological sleep. During NREM sleep, increased hippocampo–cortical synchrony and increased δ- and σ- electroencephalographic activity are responsible for memory stabilization and processing, while a transient increase in dopamine concentration in the amygdala promotes the transition from NREM to REM sleep phase. After the transition, during REM sleep, increased cholinergic neurotransmission and increased theta activity in the amygdala promote emotional memory processing and formation of dreams, while increased synaptogenic factors contribute to the neurodevelopment of the amygdala.

Dreaming is another process intricately associated with REM sleep and the amygdala. The development of new neuroimaging techniques gave us insight into this complex activity. During the REM stage, the regulation between sleep and wakefulness is orchestrated by the reticular activation system, a neural circuit that extends from the brainstem, passes through the hypothalamus, and ultimately reaches the cerebral cortex [84]. The physiological underpinnings of dreaming sleep appear to be governed by a brainstem neuronal mechanism. Key features of this generator process, with significant implications for dream theory, include the periodic and automatic activation of forebrain regions [85]. Additionally, intense and sporadic activation of sensorimotor circuits, such as reticular, oculomotor, and vestibular neurons, likely influences the spatiotemporal aspects of dream imagery. Monoaminergic neurotransmitters and acetylcholine are highly active during this stage, which may help regulate various aspects of dreaming, including the vividness and emotional intensity of dreams [86]. The limbic system, particularly the amygdala, is highly active during this stage, contributing significantly to the emotional content of the dreams. In a study by De Gennaro et al., gray matter volume and microstructure were analyzed using MRI and diffusion tensor imaging. The researchers observed that a higher mean diffusivity (MD) in the left amygdala, indicating decreased microstructural integrity, was associated with shorter dream reports and lower emotional load scores. The bizarreness of dream reports showed a negative correlation with left amygdala volume and a positive correlation with right amygdala volume MD [87]. Furthermore, a subsequent study by De Gennero et al., conducted with MRI in patients with Parkinson’s disease, revealed a significant positive correlation between the vividness of dream reports and amygdala volume [88]. The amygdala is also involved in generating Ponto–Geniculo–Occipital (PGO) waves [89]. PGO waves are distinctive electrical wave patterns observed in the brain during sleep, particularly during REM sleep [90]. These waves originate in the pons, are transmitted to the lateral geniculate nucleus in the thalamus, and subsequently project to the occipital brain cortex. They play a significant role in the generation of REM sleep and are believed to be involved in the visual imagery and dream experiences that occur during this sleep stage. PGO waves are thought to be responsible for activating visual brain regions during dreaming, which corresponds to the vivid visual experiences often reported during REM sleep. The increased cerebral blood flow in both amygdaloid complexes during REM can also indicate a direct role of these structures in the emotional aspects of dreaming [91]. The summary of the amygdala-related process during sleep is presented in Figure 2.

## 4. The Amygdala as a Bridge Between Anxiety Disorders and Sleep Disturbances

Barlow defines anxiety as a future-oriented negative mood state, involving apprehensive anticipation of potential threats or dangers, and often accompanied by a perceived lack of control and heightened physiological tension due to increased vigilance [92]. When these symptoms impede normal functioning, anxiety disorders may be diagnosed. The Diagnostic and Statistical Manual of Mental Disorders, Fifth Edition (DSM-5) identifies 11 anxiety disorders, with Generalized Anxiety Disorder (GAD) being the most prevalent (3.7% lifetime prevalence across surveys) [93]. It is characterized by excessive and persistent worry about everyday issues [94]. The second most important anxiety disorder is PTSD, closely related to the functioning of the amygdala, which is triggered by traumatic events and manifests as intrusive memories, nightmares, and avoidance [95]. Additionally, this list includes other disorders such as social phobia (involving fear of social interactions [96]), panic disorder (characterized by episodes of highly intense fear [97]), specific phobias, agoraphobia (fear occurring in public or crowded places [98]), selective mutism (a disorder in which an individual fails to speak in certain social situations [99]), or anxiety due to another medical condition. The prevalence of anxiety disorders worldwide is estimated at 4,4% according to the WHO (359 million people in 2021 [100]), making them the most common psychiatric conditions. They are usually diagnosed in early adulthood, more frequently in women. High comorbidity with other mental illnesses was observed, but in this article, we will explore their connection with sleep disturbances [101].

### 4.1. Amygdala and Its Role in Anxiety Disorders

As previously highlighted, the amygdala is pivotal in emotional processing, making it a focal point of extensive research spanning numerous years due to its potential critical involvement in anxiety disorders. Multiple studies have consistently demonstrated hyperactivation in this region, indicating a shift in the excitatory/inhibitory balance towards excitation when exposed to anxiogenic stimuli. For instance, using fMRI, Birbaumer et al. observed heightened amygdala responsiveness in patients diagnosed with social anxiety disorder when presented with neutral human faces [102]. Moreover, various investigations have shed light on the role of the amygdala during anxiety-inducing situations. Notably, Nitschke et al. revealed heightened anticipatory activity in the bilateral dorsal amygdala among individuals with generalized anxiety disorder before exposure to both aversive and neutral stimuli, distinguishing them from healthy counterparts [103]. The studies involving amygdala alteration in functional connectivity in anxiety disorder patients show that it is primarily associated with prefrontal cortex–amygdala coupling dysfunction. This circuit, crucial for automatic reactions to relevant stimuli and regulatory responses, is identified by Kim et al. as predicting higher anxiety levels associated with weakened connectivity between the amygdala and the prefrontal cortex [104]. Notably, Hahn et al. found reduced connectivity between the left amygdala and left medial orbitofrontal cortex in patients with social anxiety disorder. Heightened stress levels and a temporary decrease in negative functional connectivity between the amygdala and cortical regions mark anticipation of a social challenge in these individuals. These changes correlate with symptom severity [105]. In PTSD, amygdala responsiveness is positively linked to symptom occurrence, while the medial prefrontal cortex exhibits volumetric reduction and decreased responsiveness during symptomatic states and emotional cognitive tasks [106]. Additionally, a study by Birn et al. emphasizes diminished functional connectivity between the dorsolateral prefrontal cortex (dlFPC) and amygdala regions in anxious children with elevated temperamental anxiety [107].

Despite the extensive knowledge about alterations in the amygdala among individuals with anxiety, there remains an ongoing quest to uncover the underlying origins of these changes. The neuroinflammatory theory of mood disorders, including anxiety disorders, posits that the compromised emotional regulation observed in patients may arise from inflammation occurring both peripherally and within the central nervous system. Anxious individuals exhibit significantly elevated levels of inflammatory markers, including interleukin-1β (Il-1β), interleukin-6 (IL-6), C-reactive protein (CRP), and Toll-like receptor 4 (TLR-4), compared with their healthy counterparts [108,109]. Furthermore, disruptions in the blood–brain barrier have been observed, facilitating the influx of peripheral cytokines [110]. These circulating particles can impact the amygdala through various mechanisms. In a notable study, Tumor necrosis factor-α (TNF-α) was found to increase AMPA receptor-mediated glutamatergic transmission while concurrently decreasing GABA A receptor-mediated transmission [111]. Additionally, recent investigations by Zhong have revealed that neonatal inflammation, characterized by persistent downregulation of Transforming Growth Factor Beta 1 (TGF-β1), leads to decreased GABA-R expression in the BLA. This, in turn, induces an imbalance in the local excitation–inhibition circuits, giving rise to an anxiety-like phenotype in adult mice [112]. Furthermore, elevated interleukin 33 (IL-33) levels in the BLA were associated with reduced BDNF and impaired neurogenesis [112]. Furthermore, an additional study investigating LPS-induced anxiety models revealed a significant upregulation of IL-33 expression in the BLA. In this context, IL-33 overexpression in astrocytes was found to inhibit BDNF expression via the NF-κB signaling pathway, thereby influencing GABA synaptic transmission. This cascade of events contributed to the development of anxiety disorders [113].

Stress is another factor that contributes to the development of anxiety. In a rodent anxiety model induced by chronic restraint stress, dysregulation occurs in BLA projection neurons that receive monodirectional inputs from the dorsomedial prefrontal cortex (dmPFC → BLA PNs), with a shift in the excitatory–inhibitory balance towards excitation [114]. Given the prefrontal cortex’s role in complex mental processes, it is hypothesized that its improper control over the amygdala may contribute to situations where individuals with anxiety disorders struggle to assess stimuli accurately, leading to higher excitation levels and heightened anxiety responses even when the stimulus is not threatening. Additionally, stress can trigger the production of low-grade inflammatory markers, which may, as stated earlier, dysregulate the function of the amygdaloid complex [115].

The extensive research on the amygdala’s role in anxiety disorders reveals how crucial this structure is in its pathogenesis.

### 4.2. Sleep Disturbances in Anxiety Disorders

While sleep disturbances commonly co-occur with anxiety disorders, only GAD incorporates them into its diagnostic criteria. Up to 75% of patients with GAD experience insomnia [116]. This sleep disorder is often connected with excessive worries, but notably, it does not correlate with the severity of GAD. Moreover, a meta-analysis of subjective (self-reported) and objective (polysomnography) research has shown that anxious patients experience less sleep continuity, more subjective sleep disturbances, and a loss of over 20 min in subjective total sleep time (TST) [117]. Furthermore, the phenomenon of sleep misperception seems to be a common feature in anxiety disorders. It is defined as discrepancies between objective and subjective measures of sleep. Patients with anxiety disorders can perceive their sleep as much less satisfying than people with similar objective sleep parameters. Additionally, the development of actigraphy, a method for recording and analyzing movement by small, computerized devices worn on the body, has yielded new insights into sleep patterns in anxiety disorders [118]. Luik et al. identified disruptions in the 24 h circadian rhythm in anxiety disorders, particularly in GAD, and correlated this disruption with a shorter TST [119]. Conversely, a study conducted by Koolhaas et al. found no association between sedentary time and anxiety [120], which contradicts previous meta-analyses linking sedentary behaviors to anxiety symptoms [121]. Moreover, Helgadóttir et al. also reported no differences in sedentary behavior when comparing participants with anxiety disorders to those with depression or comorbid anxiety and depression [122]. These discrepancies may stem from factors such as the limited precision of actigraphy devices and the failure of the previous study to control for crucial confounders, including disability or occupational status.

Although each anxiety disorder presents a distinct profile of sleep disturbances, GAD and PTSD are most associated with these disruptions. Increasingly, studies identify sleep disturbances as a hallmark of PTSD [123], a recognition reflected in both the DSM-5 and ICD-10 classification systems. The occurrence of nightmares in PTSD is estimated to be as high as 96%, the highest prevalence among all psychiatric conditions [124]. Additionally, recent trauma survivors exhibit this symptom more frequently than individuals with chronic PTSD [125]. Related sleep characteristics include panic awakenings with poor or no recall of dreams, which is also common in PTSD patients [126]. Additionally, periodic leg movements or disruptive nocturnal behaviors (night sweats, dysphoric dreams, vocalizations, and dream enactment) are also more common among these patients [127]. Sleep structure appears to be intricately linked to the pathophysiology of PTSD, although research findings are inconsistent. Kobayashi et al., in a meta-analysis, demonstrated that PTSD is associated with a higher percentage of stage N1 sleep and a lower percentage of Slow Wave Sleep (SWS) compared to various controls, including healthy individuals and those exposed to trauma. Furthermore, PTSD patients tend to experience reduced TST in male-oriented studies [128]. Contrarily, more recent meta-analyses confirm a decrease in SWS but report no difference in N1 sleep [129]. Inconsistencies persist, with some studies indicating reduced sleep duration, increased REM density, more frequent REM fragmentation, and increased awakenings among PTSD patients [130,131,132,133]. In contrast, others show no significant differences in polysomnography-based sleep measures [134]. These discrepancies may stem from methodological variations and confounding factors such as age, sex, or comorbid conditions, necessitating further investigation. Numerous studies also highlight the association between circadian rhythm dysregulation and PTSD pathophysiology, affecting neuroendocrine function, immune response, metabolism, and autonomic regulation [124]. This relationship appears bidirectional, with evidence suggesting that sleep and circadian disruptions may also serve as preexisting risk factors for developing PTSD [135]. Advancements in neuroimaging have contributed to new insights. For example, Germain et al. employed a modified PET technique to reveal hypermetabolism during both wakefulness and REM sleep in PTSD patients, particularly in brain regions involved in emotion regulation, such as the mPFC, amygdala, hippocampus, and thalamus [136]. Another study by Germain et al. found increased cortical activity in NREM sleep compared to wakefulness in PTSD patients. Other researchers have identified additional indicators of hyperarousal, such as nocturnal excretion of norepinephrine metabolites and heightened HPA axis activity in PTSD [136].

These findings underscore the complex and profound connection between sleep and anxiety. Future research will further elucidate this intricate relationship.

### 4.3. Role of Amygdala in Sleep Disorders

The International Classification of Sleep Disorders, Third Edition (ICSD-3), published in 2014 and revised in 2023, is widely regarded as the gold standard for clinician diagnosis [137]. It categorizes sleep disorders into six main groups: insomnia disorders, sleep-related breathing disorders, central disorders of hypersomnolence, circadian rhythm sleep–wake disorders, sleep-related movement disorders, and parasomnia. The ICSD groups sleep disorders into six major categories. In this review, the main focus will be on insomnia, as it is the most prevalent symptom of anxiety disorder [138]. However, newer studies suggest that amygdala disturbances may also be linked to other sleep problems, such as sleep-related movement disorders [139] or hypersomnolence [140].

Insomnia is a condition characterized by dissatisfaction with sleep quantity or quality, associated with difficulty initiating sleep, maintaining sleep, or early-morning awakening, with an inability to return to sleep. Objective sleep parameters, measured, for example, by polysomnography, do not need to be disturbed. According to the DSM-5, the diagnosis of insomnia necessitates that symptoms persist for a minimum of three months, and difficulty with sleep must occur on at least three nights per week [141]. Insomnia is a condition that affects up to 25.5% of society globally [142], which makes it the most common sleep disturbance. It is more prevalent among women, older adults, employed individuals, and those who report religious affiliation. Insomnia has demonstrated a significant association with a range of psychiatric disorders, including mood disorders, substance use disorders, psychotic disorders, and, above all, anxiety disorders. Both psychological and physiological factors contribute to sleeplessness. The 3P behavioral model, first introduced by Spielman et al. in 1987, is one of the most widely recognized theoretical frameworks for explaining insomnia pathogenesis [143]. According to the Spielman Model, chronic insomnia develops from three groups of factors in an individual. Predisposing factors, such as hyperactivity, personality disorder, or genetic vulnerability, make it more probable for one not to be able to fall asleep; precipitating factors, like traumatic experiences or other sources of physiological stress (evoking an episode of sleeplessness); and perpetuating factors (changing acute insomnia into chronic) [143,144].

The first group involves mostly non-modifiable genetic and personality traits. Many studies have identified hyperarousal (expressed, for example, as hypercortisolemia [145], increased heart rate [146], or elevated metabolism [147]) as a leading predisposing factor for the development of chronic insomnia. The hyperarousal theory of insomnia suggests the pivotal role of the stress system in its pathogenesis. This system is hyperreactive in individuals with personality traits such as neuroticism, introversion, or perfectionism, which are commonly listed as predisposing factors for insomnia [148]. However, alongside sleep disturbances like chronic insomnia, the human body tends to increase arousal, making it challenging to determine whether hyperarousal is an underlying cause or consequence of insomnia [149]. Additionally, traits like social inhibition and avoidance, coupled with predispositions, suggest that insomnia patients often internalize emotions rather than expressing stress outwardly. Some studies suggest that this internalization may predispose individuals to activities such as rumination and other sleep-disrupting behaviors, and ultimately to sleeplessness.

Several genes have been implicated in insomnia, including Apolipoprotein E4 (ApoE4) [150], Period Circadian Regulator 3 (PER3) [151], Clock Circadian Regulator (CLOCK) [152], and the Serotonin Transporter-Linked Polymorphic Region (5-HTTLPR) [153]. As observed, the proteins encoded by these genes play diverse roles in the human brain, ranging from regulating lipid homeostasis and serotonin metabolism to controlling circadian rhythm. The significance of the latter aspect becomes apparent in the context of individuals with anxiety disorders. Although these individuals show no significant differences in the regulation of circadian clock proteins compared with healthy controls, mice carrying a mutation in the clock protein display a notably less anxious phenotype [154]. Furthermore, the identified precipitating factors include physical or mental health conditions, unfavorable sleep environments, traumatic experiences, medication, and stress. Anxiety can substantially hinder the ability to initiate sleep, for instance, by autonomic system activation that exacerbates difficulties in falling asleep [155]. In the final category of factors, the original author of the model identified the following variables as pertinent: excessive time in bed, irregular timing of retiring and arising, unpredictability of sleep, worry over daytime deficits, multiple bouts (naps, fragmentation) of sleep, maladaptive conditioning, increased caffeine consumption, hypnotic and alcohol ingestion.

**Figure 3:**
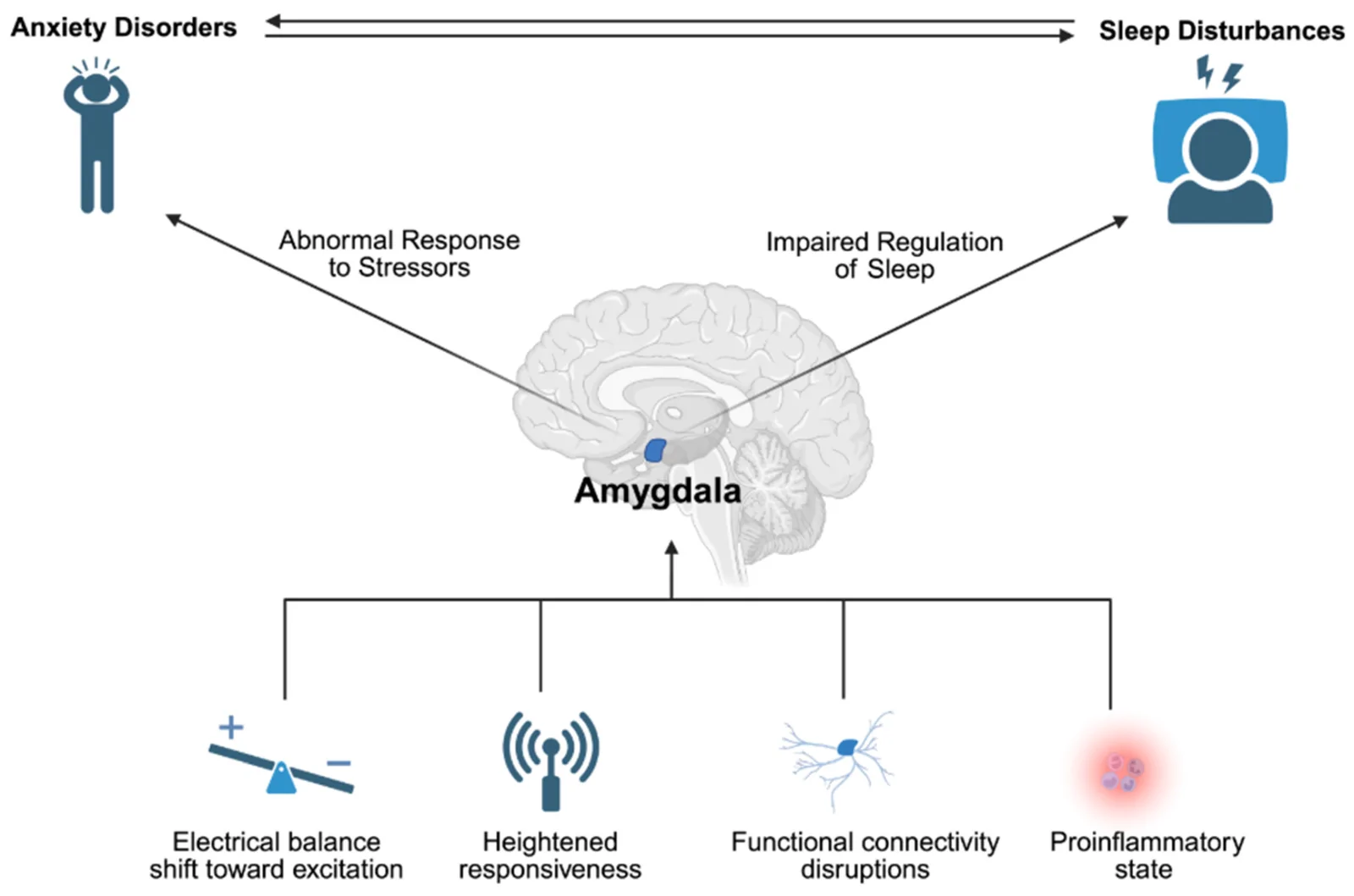
Amygdala Dysfunction in Anxiety and Sleep Disturbance. Pathophysiological alterations in the amygdala, including electrical imbalance, heightened responsiveness, disrupted connectivity with other brain regions, and proinflammatory states, contribute to its functional impairment. Given the amygdala’s central role in the pathogenesis of both anxiety disorders and sleep disturbances, such dysfunction can establish a self-reinforcing cycle: anxiety exacerbates sleep disruption. In contrast, impaired sleep further amplifies anxiety symptoms.

As the amygdala is critically involved in both insomnia and anxiety disorders, it is reasonable to hypothesize a bidirectional influence between these two disorders. Sleep deprivation leads to a functional deficit in the interaction between the amygdala and the ventral anterior cingulate cortex (ACC), contributing to a decline in mood. This deficit can also amplify the amygdala’s responsiveness to negative stimuli [156]—a mechanism involved in the occurrence and exaggeration of anxiety disorders. In a study by Prather et al., a predictive relationship was observed between amygdala reactivity to fearful facial expressions, greater depressive symptoms, and higher perceived stress in individuals with poor sleep quality, but not in those with good sleep quality [157]. Furthermore, a recent study revealed dysfunction in amygdala function among individuals with primary insomnia. Huang et al. demonstrated a significant decrease in functional connectivity, particularly between the amygdala and the insula, along with heightened connectivity with the premotor and sensorimotor cortices [158]. Conversely, connectivity with the striatum and thalamus increased. This altered functional connectivity in neural circuits responsible for emotional processing may increase an individual’s responsiveness and help explain the heightened amygdala sensitivity to insomnia-related stimuli observed by Baglioni et al. in individuals with insomnia [159]. The new neuroimaging technique enabled us to investigate amygdala abnormalities in patients with chronic sleeplessness more precisely. The Integrated Registration and Segmentation Tool, developed by the Oxford Center for fMRI of the Brain, was utilized to examine volumetric and surface alterations in patients with chronic insomnia. The analysis revealed atrophic changes in the left amygdala, specifically in the SF and BLA nuclei. The atrophic changes were also observed in the BLA of the right amygdala. The BLA nuclei are responsible for environmental information processing and self-relevant information, while the SF nuclei are highly tuned to social information processing [160]. Scientists speculated that the observed amygdala atrophy in the SF and BL nuclei might reflect abnormalities in environmental information processing and self-relevant cognition [161]. The presented findings show that the function, reactivity, and morphology of the amygdala are disrupted in insomnia, suggesting that the impact of negative stimuli is increased and prolonged in this sleep disorder [162]. Figure 3 illustrates the principal functional pathways connecting the amygdala with stress and sleep regulation.

## 5. Therapeutic Targets in the Amygdala

Presently, the foremost therapeutic approach for both anxiety disorders and sleep disturbances involves the integration of pharmacology and psychotherapy. Anxiety disorders are typically managed with a class of medications that includes selective serotonin reuptake inhibitors (SSRIs), selective serotonin and noradrenaline reuptake inhibitors (SNRIs), and benzodiazepines (administered on an ad hoc basis in states of impaired functioning, such as during panic attacks). In limited instances, atypical antidepressants, beta-blockers, or tricyclic antidepressants may be considered. To ensure the efficacy of a given substance, it is imperative to address the interconnected cycle of sleep disturbance and anxiety disorders. Without such intervention, the impaired state may persist without interruption. In the context of insomnia, the most prevalent “sleeping pills” belong to the category of nonbenzodiazepine GABA receptor agonists, commonly referred to as Z-drugs. Furthermore, innovative therapeutic options, such as agomelatine, have demonstrated effectiveness in addressing both conditions.

### 5.1. SSRIs

SSRIs help regulate amygdala function; Young et al. reported heightened amygdala activity in response to positive stimuli and decreased activity in response to negative stimuli among SSRI responders [163]. In rat models, fluoxetine treatment led to recovery from stress-induced depression and anxiety-like behavior, akin to environmental enrichment effects, broadly normalizing stress-related gene expression. Furthermore, another SSRI, escitalopram, inhibits corticotropin-releasing factor (CRF) release in the CeA nuclei. Given the anxiolytic effects of potent CRF antagonists in the amygdala, this action supports the rationale for using these drugs in anxiety treatment. The downregulation of NMDA receptors after SSRI treatment also contributed to a significant reduction in amygdala activity [164]. In pediatric subjects, escitalopram enhanced emotional processing speed and modified connectivity patterns in the amygdala, ventromedial prefrontal cortex (vmPFC), and angular gyrus during emotion processing [165]. Studies linking SSRIs to the amygdala reinforce this connection by associating treatment efficacy with various amygdala features. For example, baseline amygdala–vmPFC connectivity and escitalopram-induced increased amygdala–angular gyrus connectivity predicted subsequent improvement in anxiety symptoms [165]. Additionally, Faria et al. used PET to investigate whether amygdala–frontal couplings determine SSRI responses in social anxiety disorder. Responders and nonresponders to SSRIs and placebo exhibited different treatment-induced co-activations between the left amygdala and dlPFC, as well as the rostral ACC [166]. Conjunction analysis suggested shared anxiolysis-dependent inverse co-activations in SSRI and placebo responders between the left amygdala–dlPFC and left amygdala–rostral ACC, with a shared positive co-activation between the left amygdala and dorsal ACC. Considering the previously discussed role of the amygdala in sleep, it could be hypothesized that the observed positive alterations may lead to improved sleep in individuals who are effectively treated with SSRIs [167]. While this seems to hold in the long term, possibly also due to enhanced mood and daytime activity, the short-term findings are inconsistent and not universally observed across all members of this group of drugs. In the initial introduction of the “new antidepressant”, the authors proposed the possibility of negative impacts on sleep architecture. Specifically, fluoxetine, paroxetine, and sertraline were noted to delay the onset of REM sleep. Additionally, paroxetine was found to increase awakenings and decrease REM sleep, SWS, TST, and sleep efficiency [168]. While the effect of sertraline on sleep quality is weaker, this characteristic could present a favorable option for individuals with co-occurring anxiety disorders and insomnia [167].

### 5.2. SARIs

A similar group of drugs significantly affecting sleep is serotonin antagonists and reuptake inhibitors (SARIs), with trazodone being one of the most commonly used. Trazodone blocks 5-HT2 serotonin receptors and inhibits serotonin transporter activity, preventing serotonin reuptake. It also affects presynaptic 5-HT2 receptors and adrenergic receptors, and it blocks alpha-1 and H1 receptors [167]. These latter two actions are mainly responsible for trazodone’s influence on sleep. In a multicenter study by Walsh et al., trazodone treatment reduced the number of wakeups, increased the amount of deep sleep, and raised the proportion of N1 sleep. After seven days of treatment, participants reported longer sleep latency and reduced daytime sleepiness compared with baseline [169]. Another study by Roth et al. reported decreased awakenings, improved sleep quality, increased deep sleep, and reduced next-day sleepiness [170]. Notably, some trials have explored the use of trazodone in patients with PTSD. For instance, Hertzberg et al. utilized trazodone treatment in nine patients with PTSD. During treatment, the severity of PTSD symptoms significantly decreased, and global improvement was observed in the subjects. Importantly, this effect was sustained for 2 to 3 months and was associated with improved sleep [171]. Another research team conducted a more comprehensive analysis. In their study, which included 60 subjects who met the inclusion criteria, 72% reported that trazodone reduced nightmares, 92% reported that it aided sleep onset, and 78% reported improved sleep maintenance [172]. The recent literature reports that trazodone is successfully used not only as an antidepressant but also for individuals struggling with sleep disorders [173,174].

### 5.3. Benzodiazepines

Benzodiazepines achieve their pharmacological effects by selectively enhancing the actions of both endogenous and exogenous GABA through GABA-A receptors. This augmentation increases GABA’s apparent affinity, increasing chloride conductance without a concurrent rise in efficacy. At the single-channel level, this enhancement appears to stem from a higher likelihood of channel opening [175]. In the broader context of anxiety disorder treatment, benzodiazepines, along with other relevant drugs, target specific points within the amygdala. Early research in rats revealed that the BLA division holds the highest concentration of benzodiazepine receptors, with type 1 receptors being most prevalent [176], aligning with the amygdala’s pivotal role in anxiety generation. However, recent studies using advanced neuroimaging techniques, such as deep-brain calcium imaging or chemogenetic silencing, have shown that protein kinase C delta (PKCδ+) and somatostatin (SST-) neurons in the lateral division of the CeA are both necessary and sufficient to induce diazepam’s anxiolytic effects [177]. This discrepancy may reflect advances in neuroimaging techniques (such as deep-brain calcium imaging or chemogenetic silencing) used in recent research.

In the context of acute insomnia, short-term benzodiazepine use effectively alleviates symptoms [178]. However, due to the adverse effects associated with prolonged treatment, such as cognitive decline, an increased risk of dementia, impaired sensory and motor function, paradoxical aggressive behavior, weight gain, and the potential for addiction and withdrawal symptoms, benzodiazepines are no longer recommended as the primary pharmacological option for chronic insomnia [179]. Moreover, this class of drugs significantly disrupts sleep architecture, characterized by increased N2 sleep and decreased N3 sleep duration, as well as reduced REM sleep during nocturnal sleep. Stage N3, often described as slow-wave sleep, plays a crucial role in human physiology, particularly in learning-associated processes such as memory coding, reactivation of new memories, and memory consolidation [180]. This partly explains the adverse effects of benzodiazepines. Additionally, as previously mentioned, REM sleep is vital for emotion regulation, reinforcing the decision to avoid prescribing this drug class to individuals experiencing insomnia.

Interestingly, hypnotics like zolpidem, which also act on GABA-A receptors but with different affinity to specific subtypes, influence the amygdala but target different regions. In a study by Park et al., acute diazepam administration enhanced the potency of GABA-A receptor-mediated inhibitory postsynaptic currents (IPSCs) in the BLA and CeA subnuclei. However, zolpidem increased IPSCs with similar potency only in the BLA, and its effectiveness in the CeA was observed only at high concentrations [181]. Furthermore, Brunner et al. showed that a single dose of zolpidem significantly decreased REM sleep time in healthy individuals [182]. This study suggests that targeting specific locations in the amygdala with a drug may explain its anxiolytic and/or hypnotic effects.

### 5.4. Agomelatine

Agomelatine, a novel antidepressant approved for use in major depressive disorder in Europe and Australia, has demonstrated anxiolytic properties despite lacking approval for anxiety disorders. Studies suggest that its agonism at the melatonergic MT1 and MT2 receptors, along with antagonism at 5-HT2C receptors, contributes to its anxiolytic effects. Notably, mice lacking 5HT2c receptors exhibited attenuated fear responses [183], emphasizing the significance of this receptor subtype. Additionally, agomelatine’s capacity to antagonize 5-HT2C receptors, particularly within the amygdala and hippocampus, appears intricately linked to its anxiolytic effects. Notably, 5-HT2C blockade elevated extracellular noradrenaline levels in the hippocampus, which was shown to decrease anxiety [184]. The anxiolytic action is further supported by activation of melatoninergic receptors, as demonstrated by melatonin’s anxiolytic properties, including preoperative anxiolysis in humans and reduced fear conditioning in mice [185]. This multifaceted pharmacological profile underscores the potential of agomelatine as a promising candidate for anxiety-related conditions. Meta-analyses show that agomelatine is a well-tolerated and effective option for treating GAD [186]. In other conditions, the consensus on agomelatine efficacy is less uniform; however, a limited yet suggestive body of research proposes its potential utility in disorders such as panic disorder, social phobia, and PTSD [187,188,189].

As agomelatine is associated with improved sleep quality, it is also an interesting option for patients presenting with sleep disturbance. In a study by Quera-Salva, agomelatine significantly improved subjective symptoms of sleep–wake cycle disturbance, including sleep onset, sleep quality, and daytime alertness, as early as 1 week in randomized trials, compared with SSRIs and SNRIs. Administering agomelatine to depressive patients over six weeks resulted in a notable extension of NREM sleep while leaving REM sleep unaffected. This outcome contributed to enhancements in both the quality and continuity of sleep among the treated individuals [190]. Moreover, Grosshans et al. showed that agomelatine was effective in reducing insomnia in abstinent alcohol-dependent patients [191]. Given that this drug is not prone to abuse, it holds promise as a valuable addition for treating insomnia associated with alcohol dependence. However, agomelatine is contraindicated in patients with liver dysfunction, which is a severe limitation for agomelatine therapy in this group of patients. The influence of the above-mentioned drug groups on the amygdala, anxiety, and sleep is summarized in Table 2.

## 6. Conclusions

The amygdala is not only crucial for emotional processing and fear conditioning but also contributes to sleep architecture, dreaming, and other mental processes during sleep. Our article underscores the importance of considering the physiology and pathology of the amygdala to comprehensively grasp the co-occurrence and bidirectionality of sleep disorders and anxiety. Furthermore, when developing new drugs, it is essential to consider their potential impact on the amygdala, given its pivotal role in these interconnected processes.

Future research in this field is crucial for a comprehensive understanding of the amygdala’s established roles and the potential discovery of new ones. This knowledge can serve as a foundation for the development of new pharmacological options for people struggling with anxiety and sleep problems, which severely affect not only the quality of an individual’s life but also decrease productivity at work. Considering the worldwide prevalence of these conditions and the efficacy of current methods, such breakthroughs have the potential to provide much-needed relief to a substantial number of individuals.

## Figures and Tables

**Table 1:** Functional organization of amygdaloid nuclei.

Groups of Nuclei	Subdivision	Role
Basolateral	Basal Nuclei Lateral Nucleus Accessory Basal Nucleus	Fear Conditioning [4] Integration of signals from different modalities [5] Avoidance Behaviors [6]
Superficial	Cortical Nuclei Nucleus of the lateral olfactory tract	Detection of Incentive Social Signals [7] Increased social alertness in Response to danger [7] Emotional Response to Stimuli [7]
Centromedial	Medial Nuclei Central Nuclei	Negative Prediction Error Signaling [8] Aversive and Appetitive Learning [8] Reward Devaluation [8] Autonomic and Physiological Responses [9] Attention and Response Initiation [8]
Others	Intercalated Cell Masses	Processing inputs from basolateral amygdala [10] Feedforward inhibition to output nuclei like the central nucleus [10]. Emotional Learning [10]
	Amygdalohippocampal Nuclei	Sociosexual behaviors like mating, parenting, and aggression [11] Metabolic and cognitive functions [11]

**Table 2:** Psychotropic Drug Effects on Amygdala Function, Anxiety, and Sleep.

	Effect on Amygdala	Receptors/Systems Involved	Effect on Anxiety	Effect on Sleep
**Selective** **Serotonin Reuptake** **Inhibitors**	– Increased response to positive stimuli, decreased to negative ones – Stress-related gene expression normalization – Inhibition of CRF release in Amygdala – Increased amygdala–angular gyrus connectivity and altered (inverse and positive) co-activations between the amygdala and prefrontal regions (vmPFC, dlPFC, and ACC) during emotional processing	– CRF (inhibited) – NMDA receptors (downregulated) – Serotonergic pathways	– Reduction in anxiety- and depression-like symptoms – Supported anxiolytic action (partly similar to CRF antagonists)	– Short-term: inconsistent, sometimes disruptive effects (delay of REM onset). Increased awakenings, reduced REM, SWS, total sleep time (TST), and sleep efficiency) – Long-term: possible improvement in sleep (secondary to mood and anxiety improvements)
**Serotonin** **antagonist** **and reuptake inhibitors**	– Presumably indirect amygdala modulation (similarly to SSRIs)	– 5-HT2 receptor (antagonism) – Alpha-1 adrenergic receptor (antagonism) – Histamine H1 receptor (antagonism)	– Decrease in PTSD symptom severity – Reduction in nightmares – Overall anxiolytic and stabilizing effects, particularly in trauma-related disorders	– Better sleep continuity – Increase in deep sleep and N1 proportion – Decreased daytime sleepiness – Improved sleep onset
**Benzodiazepines**	– GABAergic-mediated activity inhibition – Highest concentration of benzodiazepine receptors in basolateral part, especially type 1 receptors – Increased GABA receptor–IPSCs in both BLA and CeA.	– GABA-A receptors (agonism) – PKCδ+ and somatostatin SST- neurons in the lateral division of the CeA	– Potent acute anxiolytic effects	– Short-term: effective relief of acute insomnia symptoms. – Long-term: – Altered sleep architecture – (increased N2 sleep, decreased N3 and REM)
**Zolpidem**	– Increased GABA receptor IPSCs in BLA, in CeA in high concentrations	– GABA-A receptors (positive allosteric modulator)	– Non-anxiolytic effect – Sedation	– Reduction in REM sleep – Lack of effect on other sleep stages
**Agomelatine**	– Antagonism of 5-HT2C receptors in the amygdala linked to anxiolytic effects. – Reduction in fear responses (shown in knockout mice). – Agonism at MT1/MT2 melatonergic receptors supports anxiolytic and fear-reducing effects.	– MT1, MT2 (agonist). – 5-HT2C (antagonist). – Noradrenergic system (increased extracellular NA via 5-HT2C blockade).	– Anxiolytic properties in GAD – Suggestive efficacy in panic disorder, social phobia, and PTSD.	– Improved sleep onset, sleep quality, and daytime alertness – Prolonged NREM sleep while REM sleep remains unaffected – Better continuity and restorative quality of sleep.

Shared pathogenetic pathways in sleep and anxiety disorders open up the possibility that new drugs and treatments for one condition could also benefit the other. Studies in mice have shown that blocking receptors in the amygdala, such as Neuropeptide Y Y(2)R in the CeA [192] or GABA receptors in the BLA [193], leads to a reduction in anxiety-like behaviors. Hypothetically, modulation of these mechanisms may also hold therapeutic potential for insomnia. Furthermore, interventions aimed at restoring amygdala physiology—through the reduction in neuroinflammation, enhancement of local function, and normalization of connectivity with other brain regions—may hypothetically reduce the comorbidity between these disorders.

## Data Availability

No new data were created or analyzed in this study. Data sharing is not applicable to this article.

## Abbreviations

ACC	Anterior Cingulate Cortex
AMPA	Alpha-amino-3-hydroxy-5-methyl-4-isoxazoleprop
ApoE4	Apolipoprotein E4
BDNF	Brain-derived neurotrophic factor
BLA	Basolateral Amygdala
CeA	Central Amygdala
CLOCK	Clock Circadian Regulator
CRF	Corticotropin-Releasing Factor
D1	Dopamine Receptor 1
dlPFC	Dorsolateral Prefrontal Cortex
DmPFC	Dorsomedial Prefrontal Cortex
DSM-5	The Diagnostic and Statistical Manual of Mental Disorders, Fifth Edition
EEG	Electroencephalography
fMRI	Functional Magnetic Resonance Imaging
GABA	Gamma-aminobutyric Acid
GAD	Generalized Anxiety Disorder
HPA	Hypothalamus–Pituitary–Adrenal
ICSD	International Classification of Sleep Disorders
IPSCs	Inhibitory Postsynaptic Currents
MA	Medial Amygdala
MD	Mean diffusivity
MG	Medial Geniculate
NMDA	N-Methyl-D-aspartate
NREM	Non-Rapid Eye Movement Sleep
N1	NREM stage 1
N2	NREM stage 2
N3	NREM stage 3
PER3	Period Circadian Regulator 3
PET	Positron Emission Tomography
PGO	Ponto–Geniculo–Occipital wave
PTSD	Post-Traumatic Stress Disorder
REM	Rapid Eye Movement Sleep
SARI	Serotonin Antagonist and Reuptake Inhibitor
SWS	Slow Wave Sleep
SSRI	Selective Serotonin Reuptake Inhibitor
SNRI	Selective Serotonin and Noradrenaline Reuptake Inhibitor
TGF-Beta 1	Transforming Growth Factor Beta 1
TST	Total Sleep Time
VmPFC	Ventromedial Prefrontal Cortex
VST	Vertex Sharp Transients
5-HTTLPR	Serotonin Transporter Linked Polymorphic Region
